# Epidemiology of dyslipidemia in Chinese adults: meta-analysis of prevalence, awareness, treatment, and control

**DOI:** 10.1186/s12963-014-0028-7

**Published:** 2014-10-28

**Authors:** Yuanxiu Huang, Lin Gao, Xiaoping Xie, Seng Chuen Tan

**Affiliations:** School of Public Health, Central South University, Changsha, China; Outcomes Research, Pfizer Investment Co. Ltd, Beijing, China; Health Economics & Outcomes Research, IMS Health Asia Pacific, 8 Cross Street, #21-01/02/03 PWC Building, Singapore, 048424 Singapore

**Keywords:** Dyslipidemia, Prevalence, Awareness rate, Treatment rate, Control rate, Meta-analysis

## Abstract

**Background:**

Numerous epidemiology studies on dyslipidemia have been conducted in China. However, a nationally representative estimate for dyslipidemia prevalence is lacking. The aim of this study is to appraise the nationwide prevalence, awareness, treatment, and control rates of dyslipidemia in adults in China.

**Methods:**

We performed a systematic review of the related observational studies published since 2003 by searching English and Chinese literature databases. Meta-analyses were conducted in eligible studies using a random effect model to summarize the dyslipidemia prevalence, awareness, treatment, and control rates. Heterogeneity and publication bias were analyzed. Sensitivity analyses were performed to explain heterogeneity and examine the impact of study quality on the results of meta-analyses.

**Results:**

Thirty-eight papers were included for meta-analyses, with a total sample size of 387,825. The prevalence, awareness, treatment, and control rates of dyslipidemia were 41.9% (95% CI: 37.7% – 46.2%), 24.4% (95% CI: 14.4% – 38.4%), 8.8% (95% CI: 7.7% – 10.0%), and 4.3% (95% CI: 4.1% – 4.5%), respectively. The prevalence of hypercholesterolemia, hypertriglyceridemia, mixed hyperlipidemia, low levels of high-density lipoprotein cholesterol, and high levels of low-density lipoprotein cholesterol were 10.1% (95% CI: 5.8% – 16.9%), 17.7% (95% CI: 14.0% – 22.1%), 5.1% (95% CI: 3.1% – 8.2%), 11.0% (95% CI: 8.0% – 15.0%), and 8.8% (95% CI: 4.1% – 17.8%), respectively. Sensitivity analyses revealed that males had a higher prevalence of dyslipidemia (43.2%) than females (35.6%). Study samples of age 30 and above in the eastern region tended to have higher prevalence of dyslipidemia. The quality of the studies has a slight impact on the pooled estimates.

**Conclusions:**

The overall pooled prevalence of dyslipidemia in Chinese adults was estimated to be 41.9%, with males having a higher rate than females.

**Electronic supplementary material:**

The online version of this article (doi:10.1186/s12963-014-0028-7) contains supplementary material, which is available to authorized users.

## Introduction

Dyslipidemia represents one of the major risk factors for atherosclerosis affecting arteries of large and medium size and consequently causing ischemia in the brain, heart, or legs. Coronary artery disease and cerebral stroke represent the major causes of morbidity and mortality among elderly and middle aged subjects [1]. The prevalence of dyslipidemia has increased dramatically in Chinese cities with lifestyle changes over the past decade, and cardiovascular diseases have emerged as a leading cause of death in Chinese adults [2].

Surveys investigating the epidemiological data of dyslipidemia in Chinese adults have been published, but the results vary due to diverse populations of different regions and the use of different diagnosis criteria [2]. An overall appraisal of dyslipidemia epidemiology nationwide in China would potentially benefit future research and policy discussions. We therefore performed meta-analyses by a comprehensive systematic review of all available evidence to synthesize the current dyslipidemia prevalence, awareness, treatment, and control among Chinese adults.

## Methods

### Search strategy and eligibility criteria

Based on the MOOSE guideline [3], we identified epidemiological studies on dyslipidemia in Chinese adults published in English or Chinese between January 2003 and August 2013. The search strategy comprised of a search of Western electronic databases (including Medline, Embase, and CINAHL) and a search of Chinese databases (including SinoMed, CNKI, and Wanfang Data). We used the search terms “dyslipidemia”, “hyperlipidemia”, “epidemiology”, “incidence”, “prevalence”, “awareness rate”, “treatment rate”, “control rate”, “China or Chinese”, and these terms’ variants and combinations.

Eligible studies had to have reported any of the following epidemiological data related to dyslipidemia in sampled populations of Chinese subjects: prevalence, awareness rate, treatment rate, and control rate. The diagnosis criteria was based on the Chinese Guidelines on Prevention and Treatment of Dyslipidemia in Adults [2], which defined dyslipidemia as any one of the following four conditions: hypercholesterolemia (total cholesterol (TC) ≥ 6.22 mmol/L); hypertriglyceridemia (triglycerides (TG) ≥2.26 mmol/L); low levels of high-density lipoprotein cholesterol (HDL-C < 1.04 mmol/L); high levels of low-density lipoprotein cholesterol (LDL-C ≥ 4.14 mmol/L).

Dyslipidemia awareness was defined as a self-report of any prior laboratory diagnosis of dyslipidemia. Treatment was defined broadly as interventions including medication, diet, exercise, and monitoring to manage dyslipidemia. Participants were considered to have controlled dyslipidemia if their serum TC, TG, LDL-C, and HDL-C were within the normal ranges as defined above based on the 2007 Chinese guidelines.

We also applied the following exclusion criteria in filtering the identified publications:

- Non-human studies
- Non-research based publications such as press releases, newsletters, forum discussions, etc.
- Non-epidemiological studies such as basic science research on dyslipidemia.
- Studies that did not disclose when the data was collected, sample size, or denominator for each reported prevalence or rate.
- Studies that investigated specific populations such as military, prisoners, specific ethnic groups, etc.
- Studies that did not apply the diagnosis criteria published in the 2007 Chinese Guidelines on Prevention and Treatment of Dyslipidemia in Adults.

### Study identification and data extraction

We identified relevant studies by searching electronic databases, scanning reference lists, and consulting clinical experts in dyslipidemia. Additionally, studies presented at key conference proceedings were identified. Two reviewers independently examined all the titles and abstracts of the studies retrieved from the searches for potentially eligible studies, then the full text of all potentially relevant citations were obtained and independently assessed by the reviewers to confirm whether they met the inclusion criteria. The results were checked and discussed by the two reviewers to agree upon a final list of included studies.

Using a standardized and predesigned data collection form, all relevant data in each included paper were extracted by two reviewers independently. The data extracted were cross-checked and any unresolved discrepancies were referred to a third reviewer. Where necessary, inputs of a clinical expert advisor were solicited to facilitate discussions among the reviewers.

For each included study, we extracted general information (including authors, year, title, type of publication, etc.), study characteristics (including study design, population, location, diagnosis criteria, sample size, etc.), participants’ characteristics (including age, sex, type of dyslipidemia, etc.). We further recorded the numbers of people with any one type of dyslipidemia condition and reported awareness, treatment, and control rates. The data stratified by sex and dyslipidemia types, where available, were also extracted.

To inform the appropriateness of included studies in the meta-analysis and later evaluate the strength of the evidence, the two reviewers independently assessed and agreed on the quality of each included study using the quality assessment checklist for epidemiological studies [4]. The checklist assesses the quality of studies on a scale of 0 (the worst) to 18 (the best) using predefined criteria on both internal and external validities [4].

### Statistical analyses and heterogeneity test

Pooled estimates of the dyslipidemia prevalence, awareness rate, treatment rate, and control rate and their corresponding 95% confidence intervals (CI) were calculated based on the random effect model [5] and stratified by sex and age group (over 18 or over 30 years) where applicable. Heterogeneity between studies was evaluated with the Cochran’s Q test and I^2^ statistic, which describes the percentage of variation across studies (values of 25%, 50%, and 75% indicate low, moderate, and high degrees of heterogeneity, respectively) [6,7]. Subgroup analyses were performed to investigate potential sources of heterogeneity from different geographical regions and types of dyslipidemia. Publication bias was evaluated by using the funnel plots method. Furthermore, sensitivity analyses were performed to evaluate the influence of particular study on a pooled estimate, which was recalculated by omitting a study each time. Independent or paired t-tests were used as appropriate, and a significant difference was reported if the p-value was less than 0.05. All statistical analyses were performed using SPSS version 20.0 (SPSS Inc, Chicago, USA) and Comprehensive Meta-Analysis software version 2.0 (Biostat, Englewood, USA).

## Results

### Characteristics of included studies

**Figure 1:**
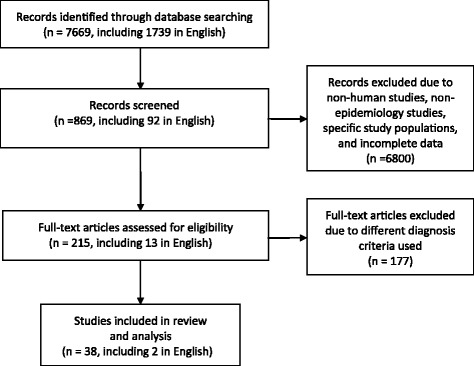
**Flow chart of screening and inclusion of studies for review and analysis.**

Our searches retrieved 7669 citations. Of these, 6800 were excluded after reading the abstracts, and 831 were further excluded after assessing the full papers, leaving 38 eligible papers for inclusion in our review and analyses (Figure 1) [8-45], which involved a total of 387,825 Chinese people.

Among the 38 included papers, 36 were written in Chinese and two were written in English; 33 studies were cross-sectional surveys and five studies employed census surveys or other designs; 18 studies were conducted in eastern China, 10 studies were conducted in central China, eight studies were conducted in western China, and two surveys crossed regions nationwide (Table 1) [8-45].

**Table 1:** **Characteristics of included studies**

**Author**	**Year**	**Region**	**Age range**	**Number of participants**	**Number of patients diagnosed with dyslipidemia**	**Score of quality assessment**
Luo R	2009	Chongqing	18-59 y	20000	6464	12
Guo MH	2011	Beijing	18-79 y	6790	3231	10
Li Y	2008	Beijing	18-79 y	2776	1269	15
Luo WP	2010-2011	Xinjiang	≥ 18 y	2999	2277	14
You AG	2007-2008	Henan	≥ 18 y	20194	-	15
Li JH	2010	National	≥ 18 y	97409	-	15
Mo J	2010	Guangdong	≥ 18 y	3577	2173	16
Pang QY	2006	Henan	≥ 18 y	984	314	9
Pang QY	2007	Henan	≥ 18 y	10127	4392	11
Li JH	2010	National	≥ 18 y	97409	51818	15
Chen YY	2010	Jiangxi	≥ 18 y	3000	1821	14
Zhang XW	2010	Zhejiang	≥ 18 y	17437	8694	14
Li XH	2007-2008	Gansu	> 18 y	3038	793	14
Hu XL	-	Zhejiang	> 18 y	2036	-	12
Li SL	2007-2008	Shanxi	20-74 y	1286	459	14
Gao B	2007-2008	Shanxi	20-74 y	3298	1106	15
Jin LZ	2007	Guangdong	20-74 y	1134	-	12
Li J	2009	Beijing	20-78 y	4332	1008	13
Wang JH	2007	Beijing	≥ 20 y	10054	-	13
Fu YY	2007	Beijing	≥ 20 y	10054	3373	16
Yin L	2008-2009	Hunan	> 20 y	1544	695	8
Fu YY	2007	Beijing	> 20 y	9786	3347	14
Guang ZJ	2011	Beijing	21-91 y	3340	993	14
Liu XY	2008	Ningxia	≥ 25 y	1275	619	14
Yuan XH	-	Guangdong	> 30 y	1053	465	8
Liao XY	2010	Sichuan	35-70 y	2032	474	14
Wang CJ	-	Henan	35-78 y	16926	7480	14
Shao YQ	2008	Zhejiang	> 35 y	7194	-	15
Zhou WJ	2008-2009	Jiangsu	> 35 y	2102	922	14
Li J	2009	Shandong	> 35 y	1972	-	15
Li Y	2008	Hubei	> 35 y	9865	2794	16
Gao Y	2011	Jilin	40-70 y	1332	618	12
Liu DW	-	Henan	≥ 45 y	4779	-	12
Zhao YZ	-	Sichuan	> 45 y	200	113	11
Wu ZF	2007-2008	Tianjin	50-94 y	1424	-	11
Sheng L	2009	Beijing	> 60 y	2685	-	13
Hu XZ	2007	Henan	> 60 y	1247	347	13
Liang YQ	2011	Guangdong	> 60 y	1135	680	12

Study quality was assessed for the 38 included papers. Four papers had quality scores between eight and 10 points, 13 papers had scores between 11 and 13 points, and 21 papers had scores between 14 and 16 points. Overall study quality was upper-middle and high, and the study quality did not differ significantly among the included studies.

### Prevalence of dyslipidemia

**Figure 2:**
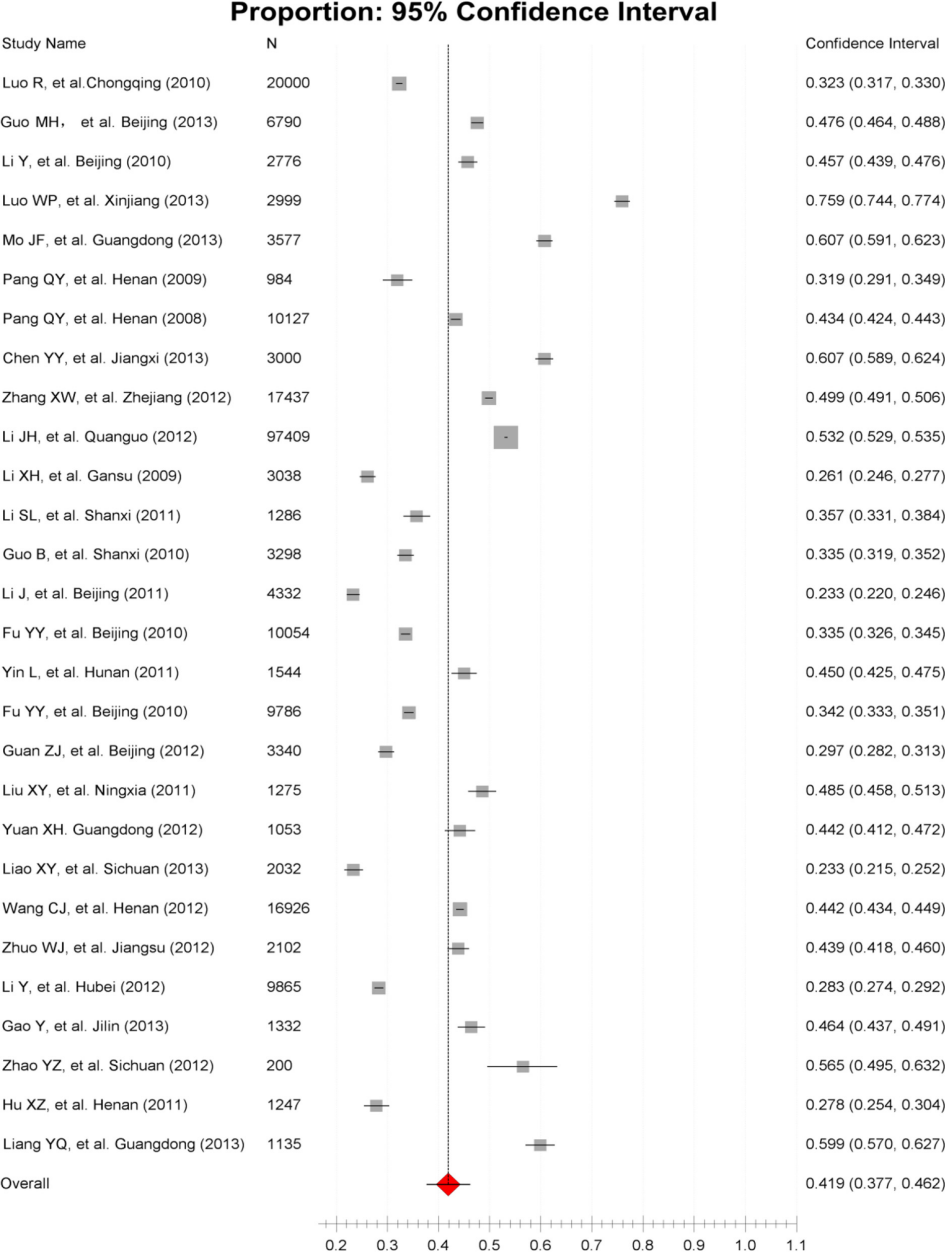
**Forest plot of 28 studies’ reported prevalence of dyslipidemia.**

Twenty-eight papers reported the total numbers of participants in the studies and those who were diagnosed with dyslipidemia (Figure 2). The pooled prevalence of dyslipidemia in Chinese adults was 41.9% (95% CI: 37.7% – 46.2%). The t-tests showed there were no statistically significant differences between subgroups stratified by age and geographic region (Tables 2 and 3); however, the pooled prevalence of dyslipidemia in males (43.2%) was higher than in female (35.6%) with statistical significance (t = 3.08, p < 0.05).

**Table 2:** **Prevelance of dyslipidemia by different age groups**

**Age range**	**Number of studies**	**Total numbers of participants**	**Median (%)**	**Minimum (%)**	**Maximum (%)**	**Pooled prevalence (%) (95% CI)**
≥ 18 y	19	203052	43.4	23.3	75.9	42.4 (37.2 ~ 47.7)
≥ 30 y	9	35892	44.2	23.3	59.9	40.9 (33.7 ~ 48.5)
Total	28	238944	44	23.3	75.9	41.9 (37.7 ~ 46.2)

**Table 3:** **Prevalence of dyslipidemia by different regions and sexes**

**Groups**		**Number of studies**	**Total number of participants**	**Median (%)**	**Minimum (%)**	**Maximum (%)**	**Pooled prevalence (%) (95% CI)**
**≥ 18 y**							
Sex	Male	15	80145	44.3	25.8	68.9	45.6 (40.0 ~ 51.4)
	Female	15	96864	33.7	14.6	56.4	35.9 (29.5 ~ 42.8)
Region	Eastern China	8	58092	40	23.3	60.7	40.0 (32.6 ~ 47.9)
	Central China	4	15655	44.2	31.9	60.7	45.1 (35.2 ~ 55.5)
	Western China	6	31896	34.6	26.1	75.9	41.9 (28.9 ~ 56.1)
**≥ 30 y**							
Sex	Male	3	5442	26.2	24.9	45.3	31.5 (20.7 ~ 44.8)
	Female	3	7772	30.2	29.9	43	34.1 (26.1 ~ 43.3)
Region	Eastern China	3	4290	44.2	43.9	59.9	49.3 (39.4 ~ 59.3)
	Central China	4	29370	36.3	27.8	46.4	36.3 (26.6 ~ 47.1)
	Western China	2	2232	39.9	23.3	56.5	38.5 (13.1 ~ 72.2)
**Total**							
Sex	Male	18	85587	43.7	24.9	68.9	43.2 (37.4 ~ 49.1)
	Female	18	104636	32.7	14.6	56.4	35.6 (30.0 ~ 41.7)
Region	Eastern China	11	62382	44.2	23.3	60.7	42.5 (36.1 ~ 49.2)
	Central China	8	45025	43.8	27.8	60.7	40.6 (33.9 ~ 47.7)
	Western China	8	34128	34.6	23.3	75.9	41.0 (30.0 ~ 53.0)

In terms of the different types of dyslipidemia, the pooled prevalence estimates of hypercholesterolemia (TC), hypertriglyceridemia (TG), mixed hyperlipidemia (TC + TG), low levels of high-density lipoprotein cholesterol (HDL-C), and high levels of low-density lipoprotein cholesterol (LDL-C) are 10.1% (95% CI: 5.8% – 16.9%), 17.7% (95% CI: 14.0% – 22.1%), 5.1% (95% CI: 3.1% – 8.2%), 11.0% (95% CI: 8.0% – 15.0%), and 8.8% (95% CI: 54.1% – 17.8%), respectively. There were no significant differences between age groups within each type of dyslipidemia condition (Table 4 and Additional file 1).

**Table 4:** **Prevelance of dyslipidemia by different dyslipidemia types**

**Types**	**Age group**	**Number of studies**	**Total number of participants**	**Median (%)**	**Minimum (%)**	**Maximum (%)**	**Pooled prevalence (%) (95% CI)**
Hypercholesterolemia (TC)	≥ 18 y	9	143350	7.7	2.5	41.8	7.9 (4.6 ~ 13.2)
	≥ 30 y	10	41366	10.8	4.9	48.1	12.5 (5.8 ~ 25.0)
	Total	19	184716	8.2	2.5	48.1	10.1 (5.8 ~ 16.9)
Hypertriglyceridemia (TG)	≥ 18 y	9	143350	15.3	4.5	36.9	13.7 (10.7 ~ 17.4)
	≥ 30 y	10	41367	18.8	11.7	44.5	22.1 (16.0 ~ 29.7)
	Total	19	184717	17.1	4.5	44.5	17.7 (14.0 ~ 22.1)
Mixed hyperlipidemia (TC + TG)	≥ 18 y	3	11249	6.5	1.9	4.2	4.8 (2.4 ~ 9.2)
	≥ 30 y	1	2102	-	-	-	-
	Total	4	13351	6.4	1.9	8.3	5.1 (3.1 ~ 8.2)
Low blood high-density lipoprotein cholesterol (HDL-C)	≥ 18 y	9	143350	6.9	0.2	57.3	11.0 (6.9 ~ 16.9)
	≥ 30 y	9	38129	10.1	1.6	32.8	11.0 (6.6 ~ 17.9)
	Total	18	181479	10	0.2	57.3	11.0 (8.0 ~ 15.0)
High blood low-density lipoprotein cholesterol (LDL-C)	≥ 18 y	5	130826	3.1	2.3	67.5	7.6 (2.1 ~ 24.3)
	≥ 30 y	7	29843	12.2	1.7	21.3	9.7 (4.5 ~ 19.8)
	Total	12	160669	8.8	1.7	67.5	8.8 (4.1 ~ 17.8)

### Dyslipidemia awareness, treatment, and control

Using the dyslipidemia awareness rates reported in four studies, we arrived at a pooled estimate of 24.4% (95% CI: 14.4% – 38.4%). Based on the reported dyslipidemia treatment rates in two studies, we estimated a pooled treatment rate of 8.8% (95% CI: 7.7% – 10.0%). There were no significant differences between males and females in terms of dyslipidemia awareness and treatment rates (Table 5). Only one paper reported a dyslipidemia control rate, which was 4.3% (3.1% for males and 5.5% for females).

**Table 5:** **Dyslipidemia awareness and treatment rates**

**Groups**	**Number of studies**	**Total number of participants**	**Median (%)**	**Minimum (%)**	**Maximum (%)**	**Pooled prevalence (%) (95% CI)**
**Awareness rate**						
Male	3	28021	25	12.4	25.3	20.1 (11.2 ~ 33.3)
Female	3	28439	31.2	14.7	32.9	25.2 (13.0 ~ 43.1)
Total	4	59807	28.9	13.5	29.8	24.4 (14.4 ~ 38.4)
**Treatment rate**						
Male	2	26620	7.2	6.8	7.6	7.6 (7.3 ~ 7.9)
Female	2	26467	10.9	9.2	12.5	10.6 (7.8 ~ 14.2)
Total	2	53087	9.1	8.4	9.7	8.8 (7.7 ~ 10.0)

### Sensitivity analysis, publication bias and heterogeneity

Two studies were assessed with a quality score of eight, the lowest among the included studies. In the sensitivity analysis, omitting these two studies resulted in a slight change in the pooled dyslipidemia prevalence estimate, 41.7% from 41.9%. The pooled prevalence of TC, TG, HDL-C, and LDL-C changed to 9.4%, 17.0%, 10.7%, and 8.3% from 10.1%, 17.7%, 11.0%, and 8.8%, respectively. Funnel plots were produced for the prevalence of each type of dyslipidemia (see the Additional file 1). Asymmetric funnels suggest the possibility of publication bias. A moderate degree of heterogeneity was observed in the pooled dyslipidemia prevalence (I^2^ = 49.9%, Q = 1.00, P < 0.001), awareness rate (I^2^ = 49.9%, Q = 0.99, P < 0.001), and treatment rate (I^2^ = 38.0%, Q = 0.721, P = 0.098).

## Discussion

Our systematic review included 38 observational studies conducted in the past decade, covering most of the regions in China, and involving a total of 387,825 participants, representing the adult Chinese population. The pooled estimates from the meta-analyses showed high prevalence of dyslipidemia and low awareness, treatment, and control rates. The study and results are timely, as currently there is a lack of published up-to-date nationwide epidemiological data of dyslipidemia to support the evidence-based approach and management of dyslipidemia in China.

The pooled estimate of dyslipidemia prevalence among Chinese adults is 41.9%, which has more than doubled in the last 10 years [46], approaching the reported prevalence of 53% from the US National Health and Nutrition Examination Survey 2003–2006 [47]. The observed increase of dyslipidemia prevalence in China could possibly be attributed to the increasingly aging population and dramatic lifestyle changes coupled with economic growth, especially changes in cigarette smoking, dietary, and alcohol drinking behaviors among the general population [48].

The study discovered that hypertriglyceridemia (TG) was the most prevalent form of dyslipidemia, with a pooled estimate of 17.7%, followed by low levels of blood high-density lipoprotein cholesterol (HDL-C) (11.0%), hypercholesterolemia (TC) (10.1%), high levels of low-density lipoprotein cholesterol (LDL-C) (8.8%), and mixed hyperlipidemia (TC + TG) (5.1%). As dyslipidemia is one of the well-established risk factors for cardiovascular diseases [47], these results highlight the extensive need for appropriate interventions, both clinical and non-clinical, to treat all types of dyslipidemia. In addition, other considerations such as improving general awareness about dyslipidemia among both patients and health care professionals and promoting healthy diet and lifestyles are equally important in designing and implementing relevant public health strategies.

We calculated and compared the pooled estimates of dyslipidemia prevalence in age groups over 18 years and over 30 years. No significant difference was found between these age groups across different types of dyslipidemia conditions. However, trends of higher prevalence of TC, TG, and LDL-C abnormalities were observed in studies of older participants (≥30 years old) compared to those studies that enrolled younger participants (≥18 years old). We observed the highest estimate at 49.3% (95% CI: 39.4-59.3) among all of the pooled prevalence estimates in people over 30 years old in the eastern region of China. Another study in China concluded that greater burden of diet-related chronic conditions including dyslipidemia has been observed in economically vibrant and highly urbanized areas [49], which are predominantly in the eastern coastal region. More economically developed areas tend to have better access to health care facilities, which might have contributed to diagnosis and hence higher reported prevalence rates in these studies among populations in the eastern region of China.

The systematic review showed that dyslipidemia is more common in men than in women, which is consistent with the findings from American population-based studies [50,51]. There were no significant differences in awareness, treatment, and control rates between the groups of men and women in China. However, studies conducted in the US showed that dyslipidemia was treated and controlled less often in men than in women [50]. A possible explanation for the higher dyslipidemia prevalence observed in men could be their higher probability of cigarette smoking, alcohol, and consumption of foods high in cholesterol.

The quality of studies included in our systematic review were generally good, thus the sensitivity analysis did not demonstrate much difference in the results of meta-analyses when the studies with the lowest quality scores were removed in the analyses. We believe our searches were comprehensive, although the asymmetric funnel plots suggested the possibility of publication bias. The moderate heterogeneity observed could come from the different study settings and populations. We investigated possible sources and performed subgroup analyses, but that still inadequately explained the heterogeneity findings in this study. The recent research of heterogeneity suggested that I^2^ estimates need to be interpreted with caution when a meta-analysis only includes a limited number of events or trials, in particular when the number of studies for further subgroup analyses is small [52]. Nevertheless, these uncertainties indicate the need of a higher-quality national survey with larger samples with better represented coverage across China.

Our study has some limitations. First, all the included studies were published after 2007 as we used the Chinese Guidelines on Prevention and Treatment of Dyslipidemia in Adults, published in 2007, as the diagnosis criteria [2]. Studies published before 2007 were based on a different set of diagnosis criteria, and therefore have not been included in our meta-analyses. Secondly, we could not investigate the differences in dyslipidemia prevalence between rural and urban areas due to the limited information reported in the included studies. Furthermore, we would like to highlight that the meta-analysis results on awareness, treatment, and control rate need to be interpreted with caution, as the number of studies reporting the relevant data are relatively small.

## Conclusions

The pooled estimate of dyslipidemia prevalence among Chinese adults was 41.9%, and hypertriglyceridemia (TG) is the most prevalent dislipidemia condition. The prevalence of dyslipidemia in males was higher than in females. Our study also showed low dyslipidemia awareness, treatment, and control rates in China.

## Additional file

Additional file 1:
**Forest plots and funnel plots of different sets of analyses are available as appendices at** ***PHM*** **online.**
