# Supplementary material for: Epidemiology of dyslipidemia in Chinese adults: meta-analysis of prevalence, awareness, treatment, and control
Source: Popul Health Metr. 2014 Oct 28;12:28. doi: 10.1186/s12963-014-0028-7 (PMC4219092; doi:10.1186/s12963-014-0028-7)
Supplement: Additional file 1: — Forest plots and funnel plots of different sets of analyses are available as appendices at PHM online. [file 12963_2014_28_MOESM1_ESM.doc]

**Additional file 1 Forest plot of** **19 studies reported hypercholesterolemia (TC)**

**
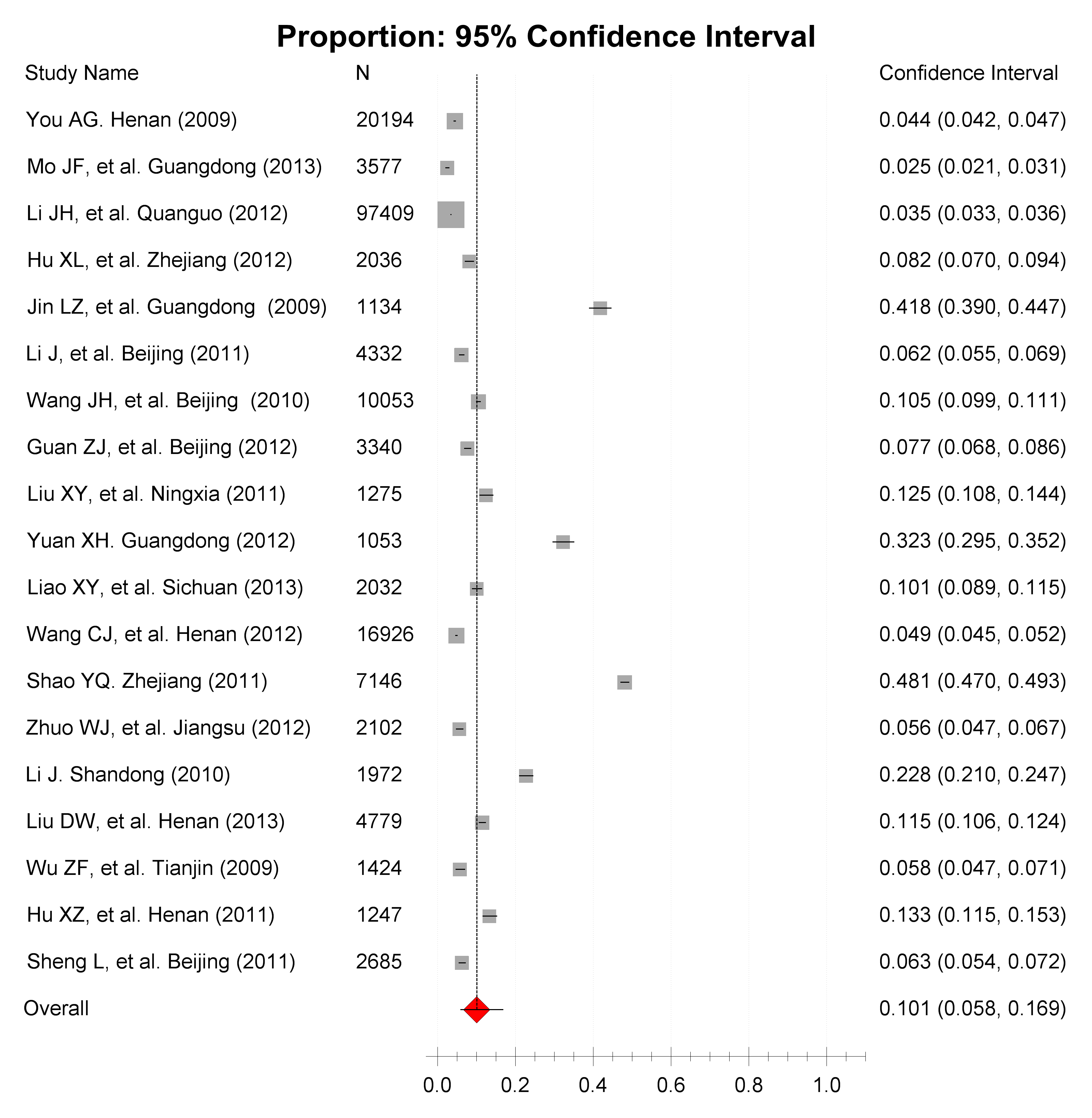
**

**Appendix 2. Forest plot of** **19 studies reported hyperglyceridemia (TG)**


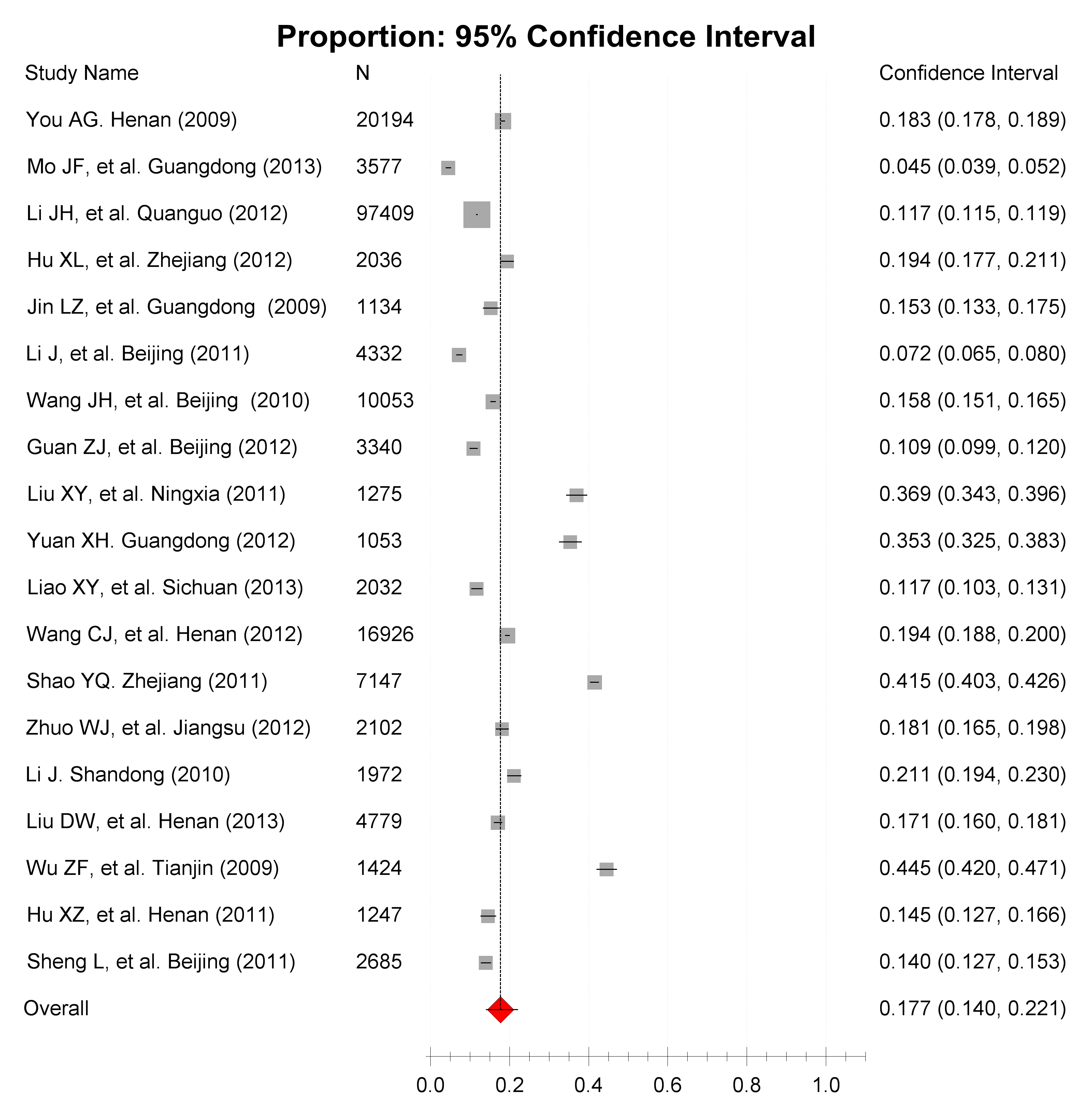


**Appendix 3. Forest plot of** **4 studies reported mixed hyperlipidemia (TC+TG)**


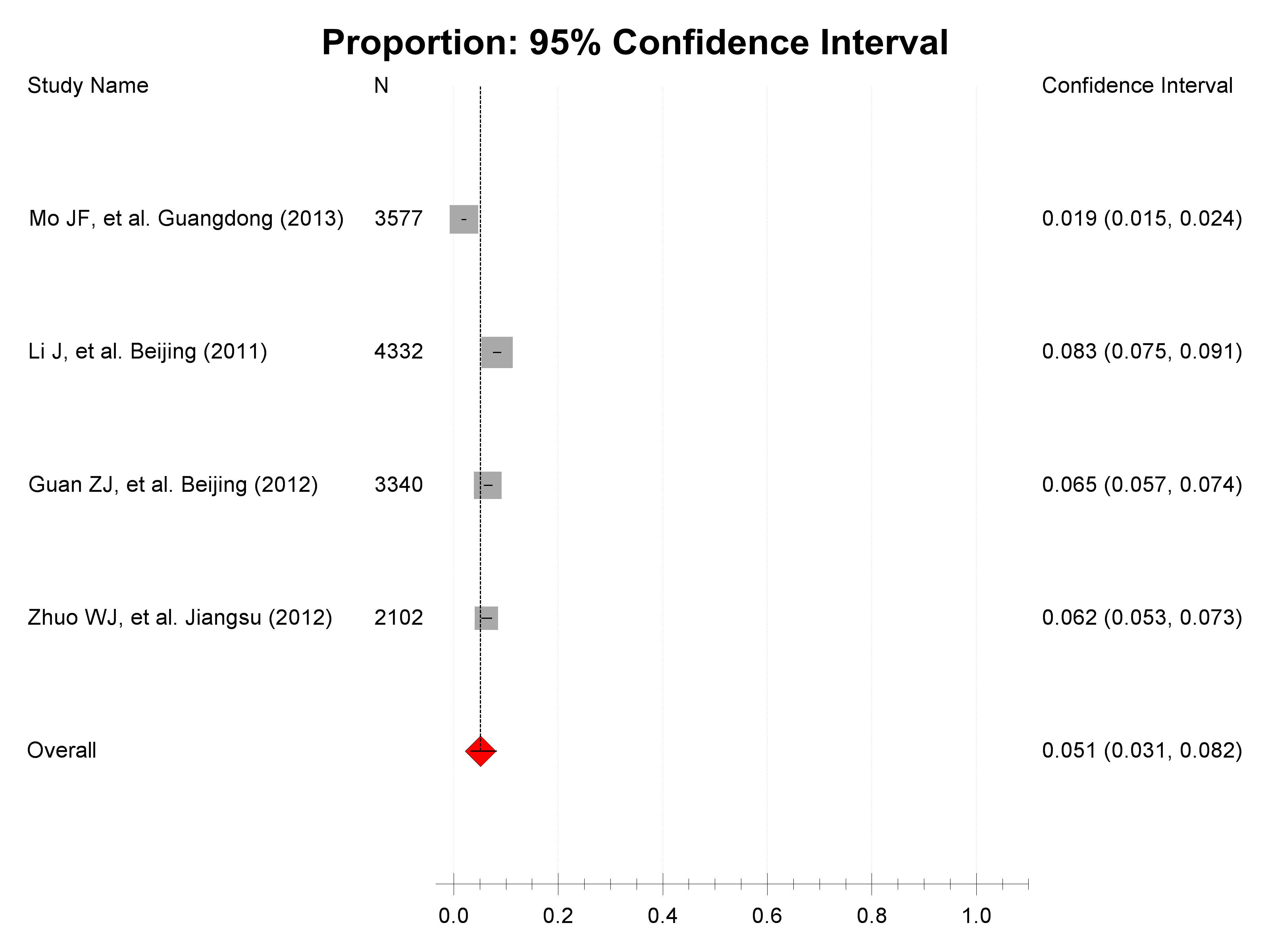


**Appendix 4. Forest plot of** **18 studies reported low level of high density lipoprotein cholesterol (HDL-C)**


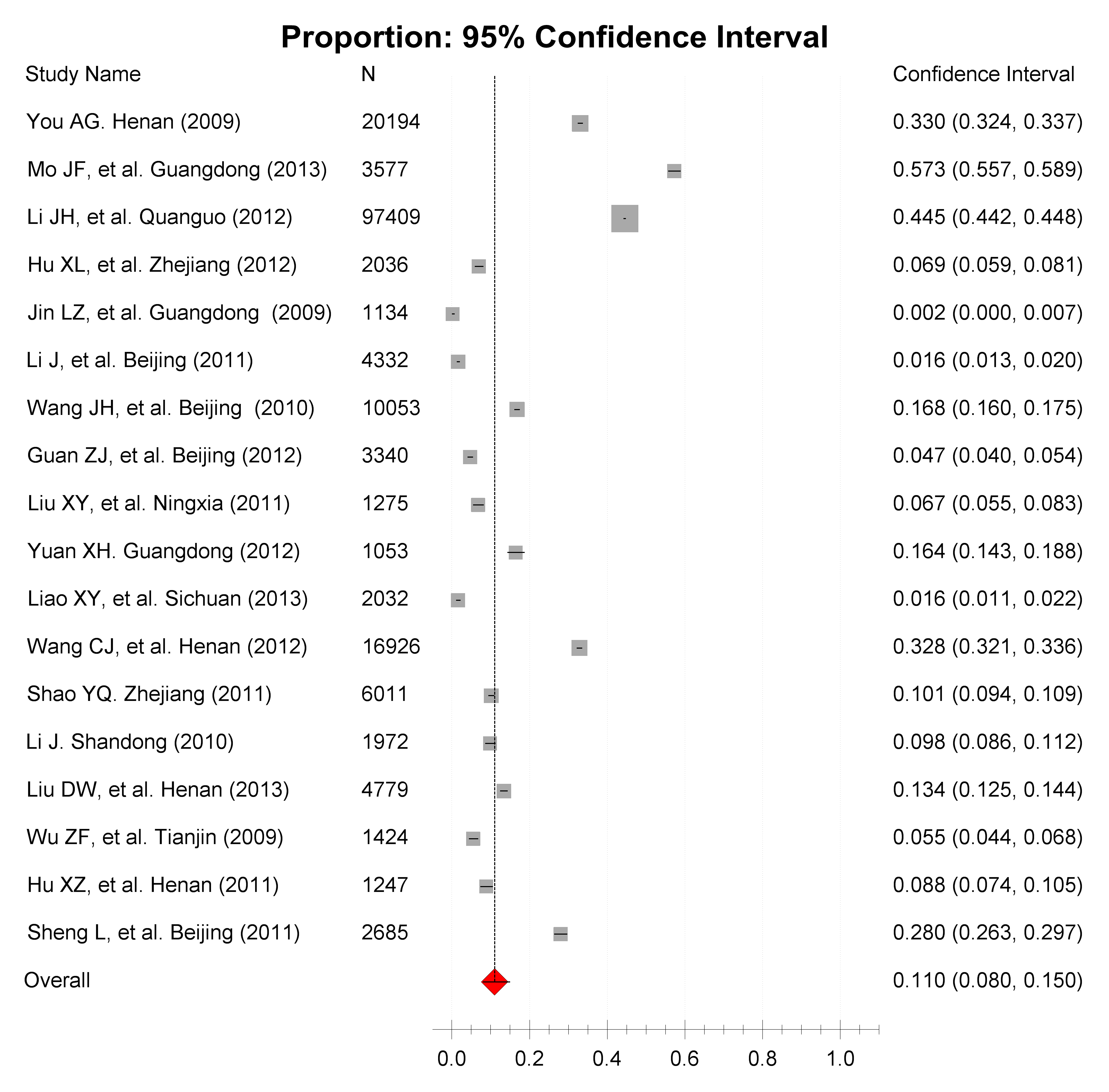


**Appendix 5. Forest plot of** **12 studies reported high level of low density lipoprotein cholesterol (LDL-C)**


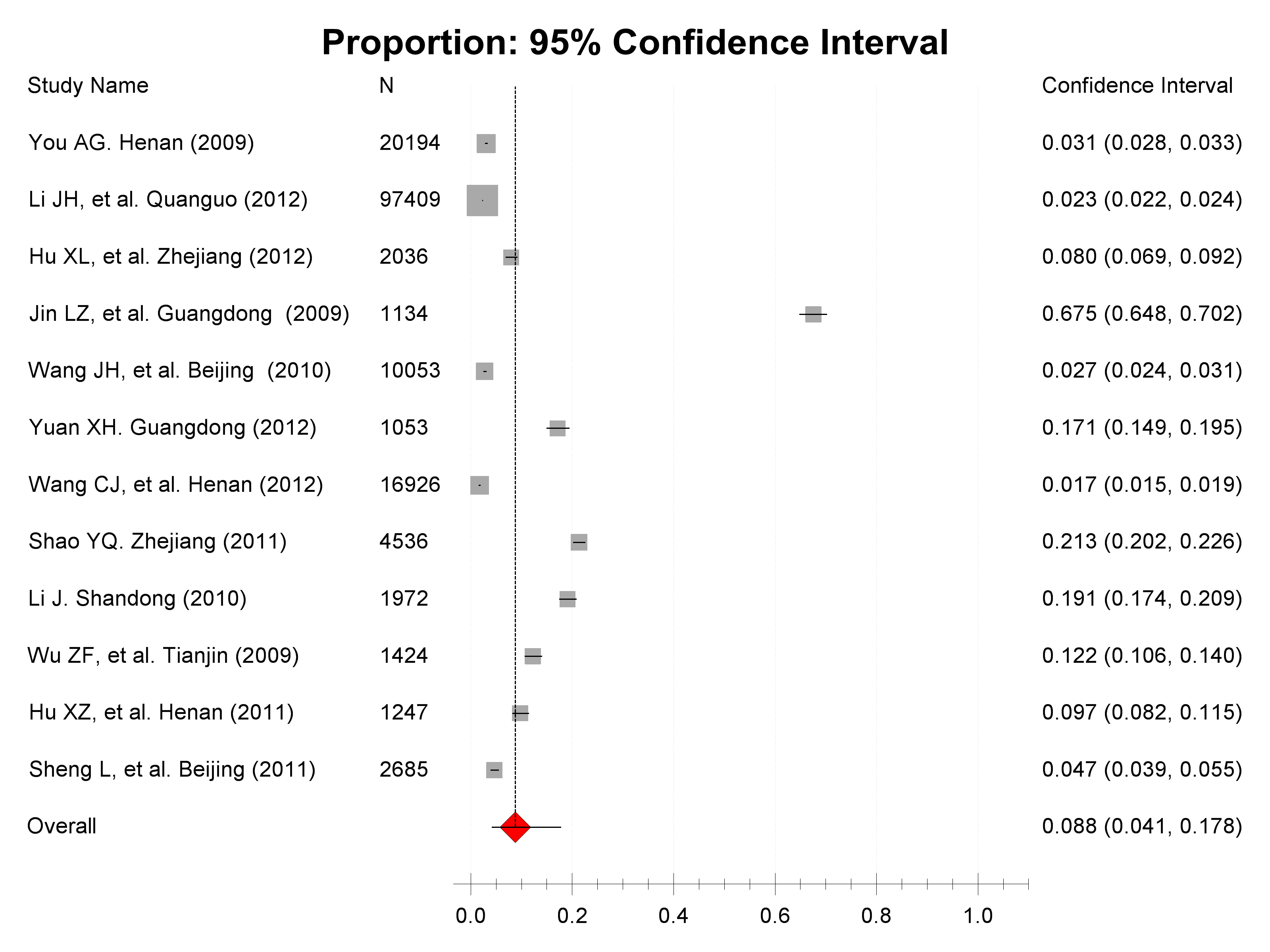


**Appendix 6. Funnel plot of 28 studies reported prevalence of dyslipidemia**

**
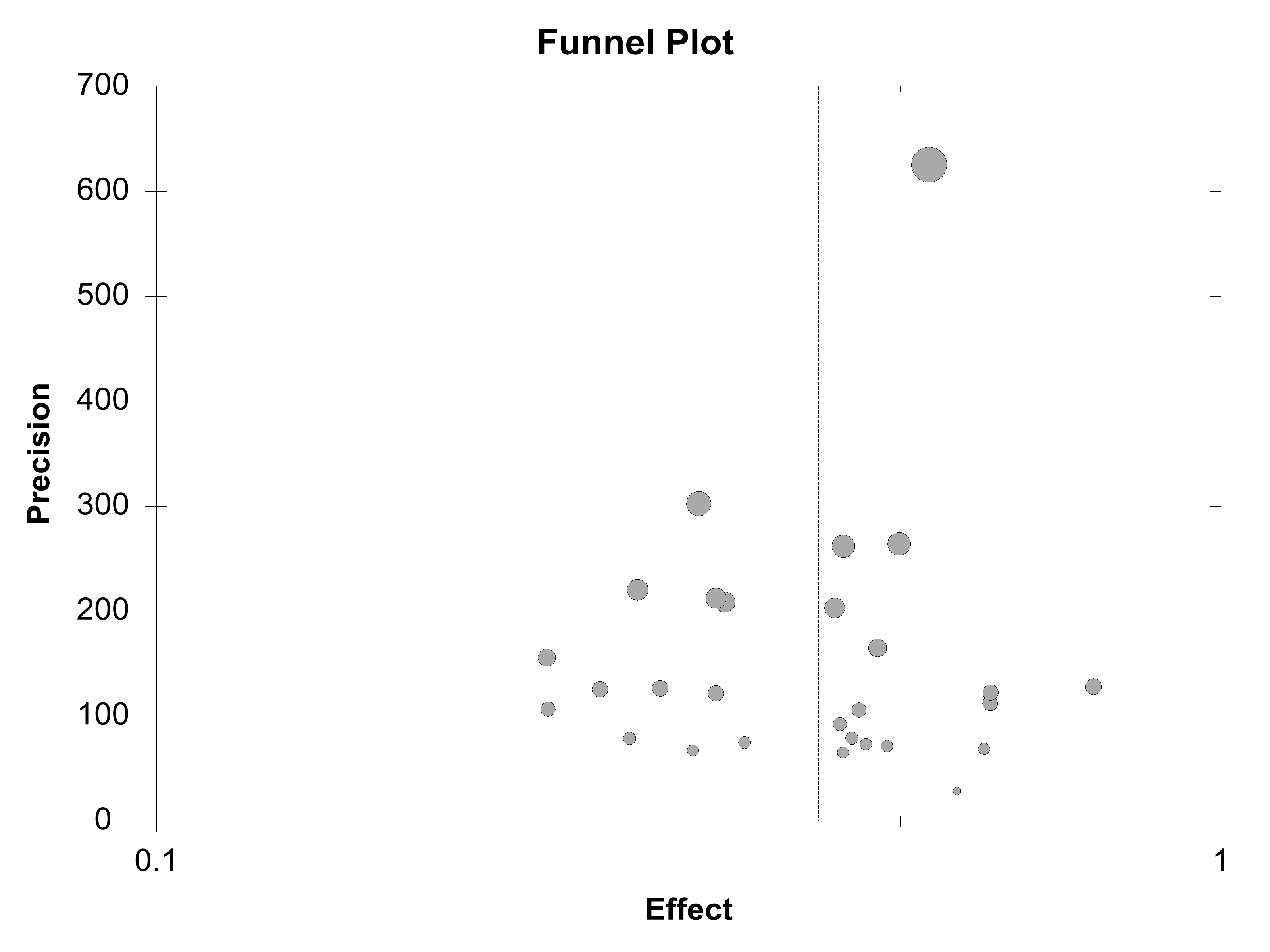
**

**Appendix 7. Funnel plot of 19 studies reported hypercholesterolemia (TC)**

**
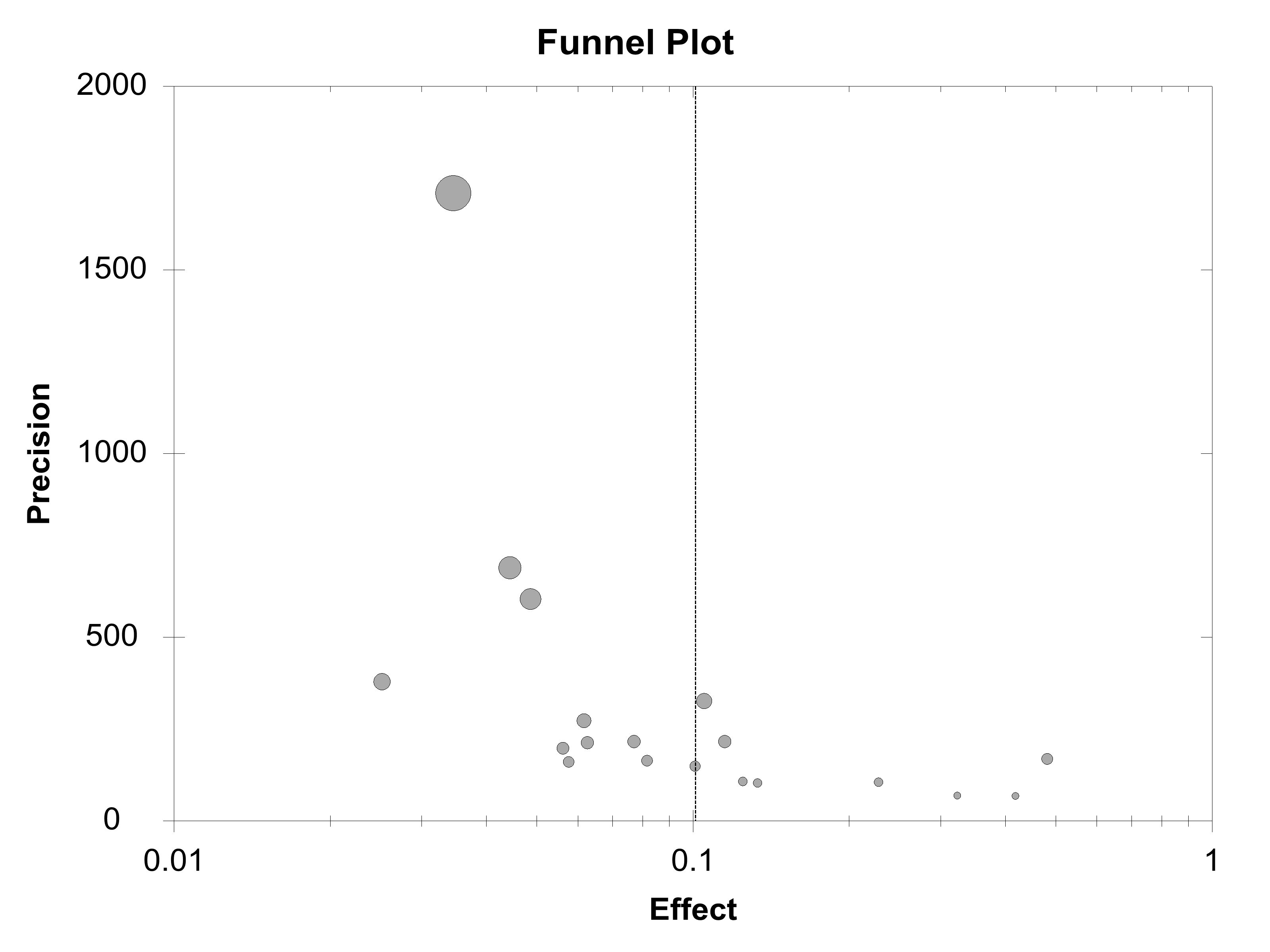
**

**Appendix 8. Funnel plot of 19 studies reported hyperglyceridemia (TG)**

**
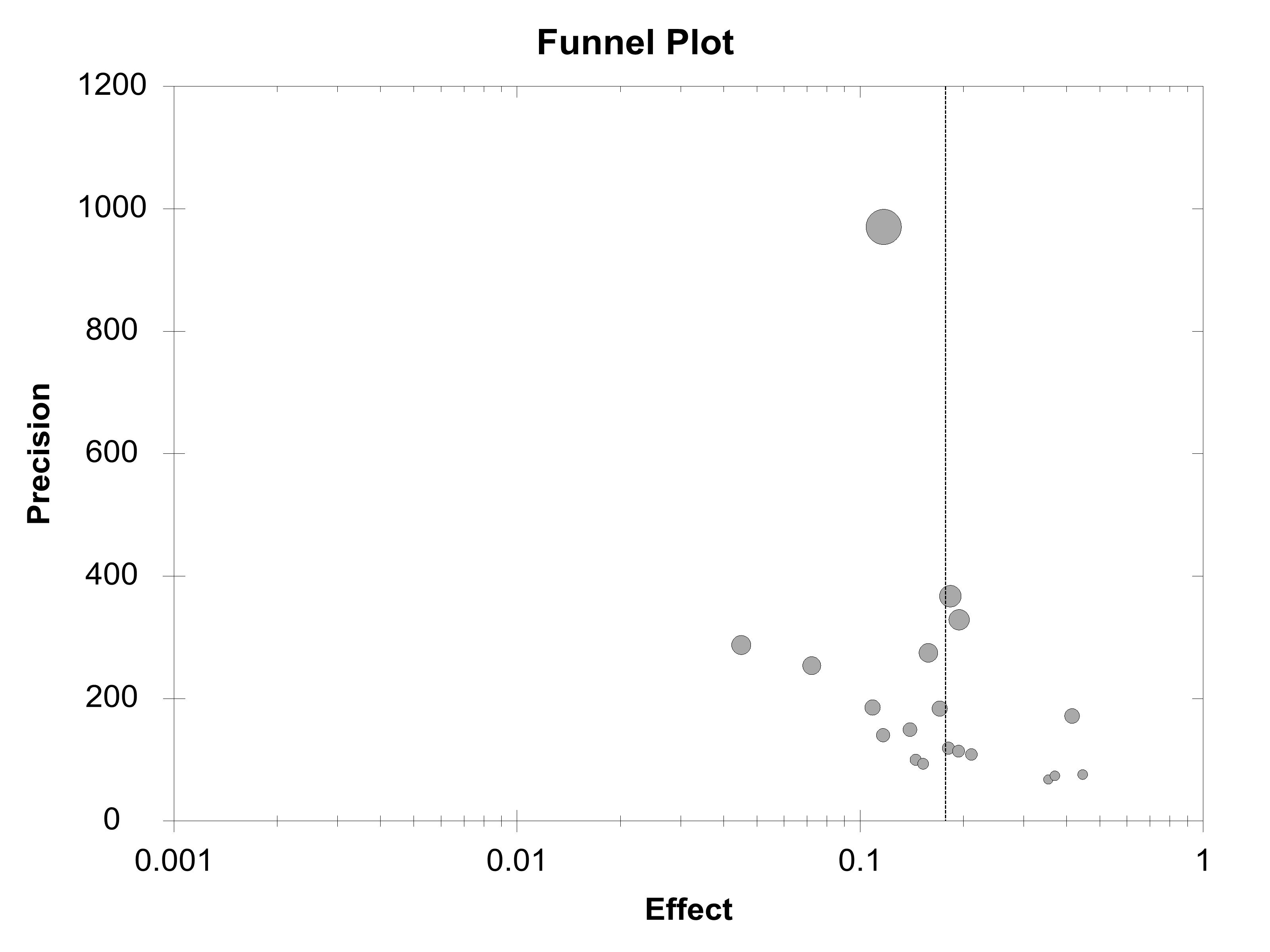
**

**Appendix 9. Funnel plot of 18 studies reported low level of high density lipoprotein cholesterol (HDL-C)**

**
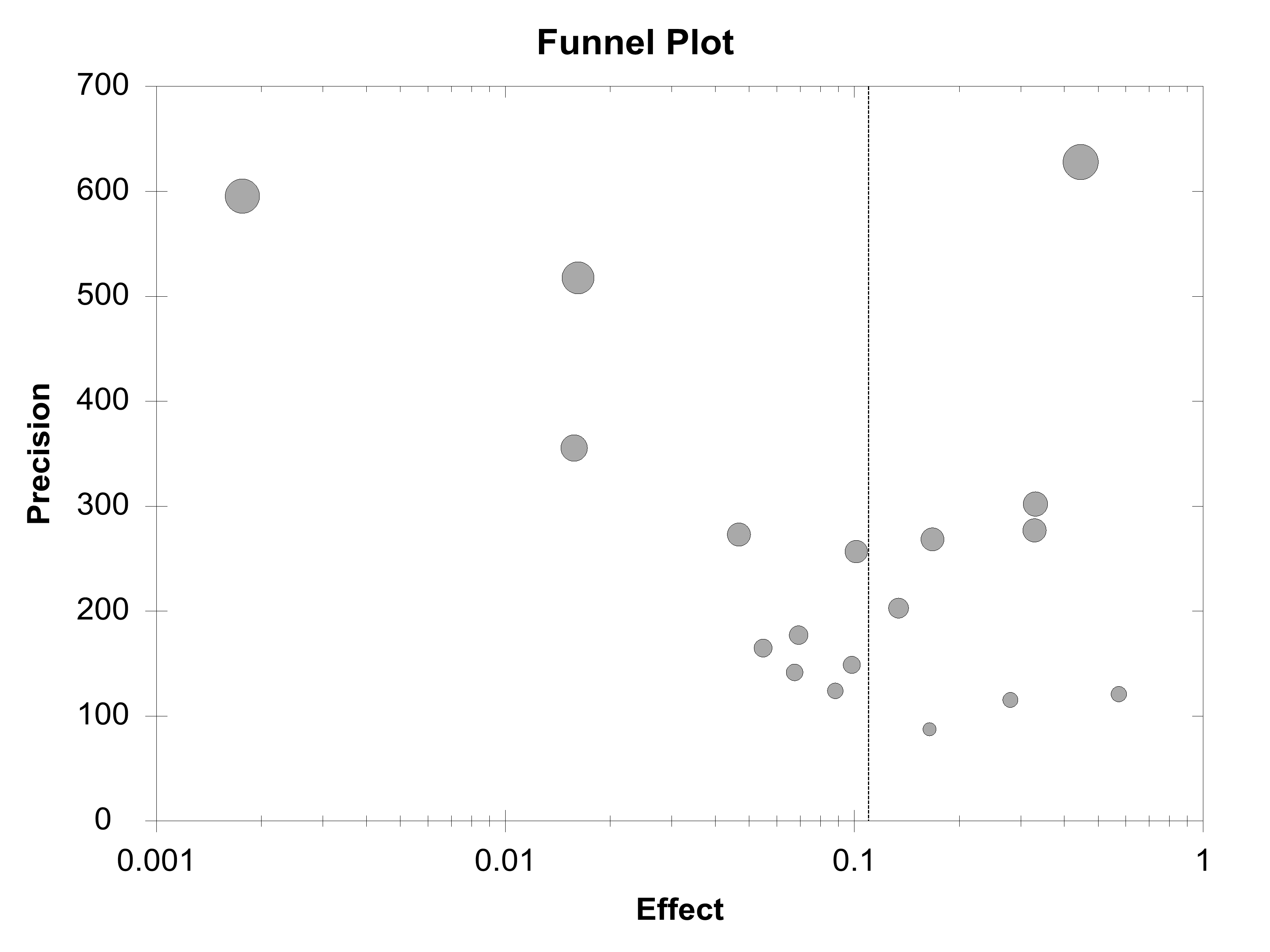
**

**Appendix 10. Funnel plot of 12 studies reported high level of low density lipoprotein cholesterol (LDL-C)**

**
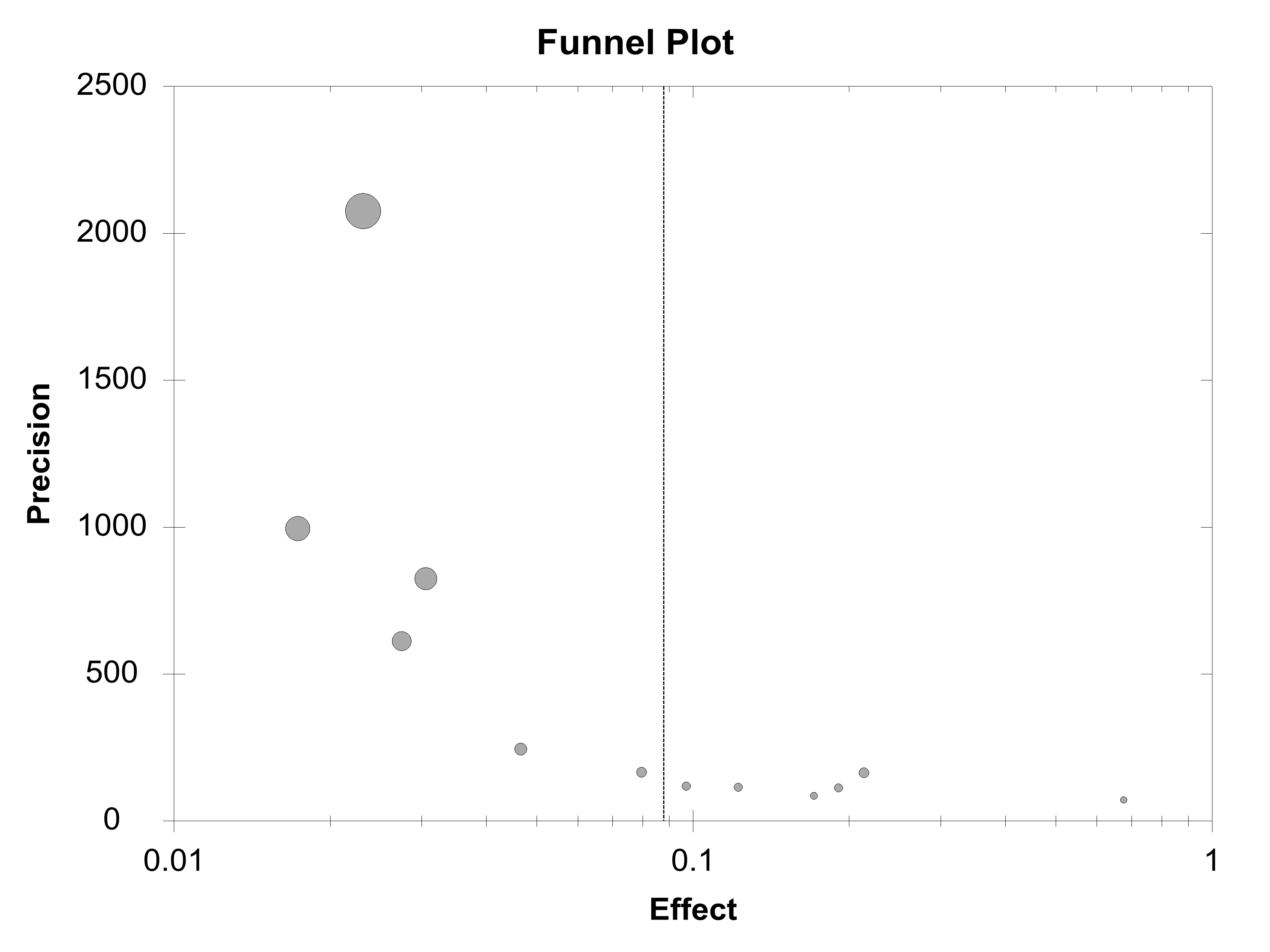
**
